# Clonotypically similar hybrid **αβ** T cell receptors can exhibit markedly different surface expression, antigen specificity and cross‐reactivity

**DOI:** 10.1111/cei.12610

**Published:** 2015-05-15

**Authors:** C. Motozono, J. S. Bridgeman, D. A. Price, A. K. Sewell, T. Ueno

**Affiliations:** ^1^ Center for AIDS ResearchKumamoto UniversityKumamotoJapan; ^2^ Institute of Infection and ImmunityCardiff University School of MedicineHeath ParkCardiffUK; ^3^ International Research Center for Medical ResearchKumamoto UniversityKumamotoJapan; ^4^ Present address: C. Motozono is currently with the Department of Immunology, Kinki University School of MedicineOsakaJapan; ^5^ Present address: J. S. Bridgeman is currently with Cellular Therapeutics Ltd, UMIC Bio‐Incubator48 Grafton StreetManchesterM13 9XXUK

**Keywords:** CD8^+^ T cells, CDR3, HIV, TCR

## Abstract

Emerging data indicate that particular major histocompatibility complex (MHC)‐bound antigenic peptides can be recognized by identical or near‐identical αβ T cell receptors (TCRs) in different individuals. To establish the functional relevance of this phenomenon, we artificially paired α and β chains from closely related TCRs specific for the human leucocyte antigen (HLA)‐B*35:01‐restricted HIV‐1 negative regulatory factor (Nef)‐derived epitope VY8 (VPLRPMTY, residues 74–81). Several hybrid TCRs generated in this manner failed to express at the cell surface, despite near homology with naturally isolated αβ chain combinations. Moreover, a substantial proportion of those αβ TCRs that did express lost specificity for the index VY8 peptide sequence. One such hybrid αβ pair gained neo‐variant specificity in the context of the VY8 backbone. Collectively, these data show that clonotypically similar TCRs can display profound differences in surface expression, antigen specificity and cross‐reactivity with potential relevance for the control of mutable viruses.

## Introduction

Engineered T cell receptor (TCR) gene transfer is a promising approach to the treatment of several conditions 1, achieving notable clinical success in patients with metastatic malignancies 2, 3. Nonetheless, outcomes vary and there is a pressing need for improvement. Various strategies have been employed to attain greater potency, including: (i) enhancement of TCR surface expression via human/murine constant region exchange 4 or insertion of an additional disulphide bond in the extracellular constant domain 5, 6; (ii) TCR affinity maturation via selection from randomly mutagenized receptor libraries 7 expressed in yeast 8, phage 9, 10, 11 or other systems 12, 13; (iii) computational design 14, 15, 16; and (iv) targeted mutagenesis within the third complementarity determining region (CDR3), which is generally responsible for peptide antigen recognition 17, 18, 19. All these efforts are linked by common aims, namely engineering TCRs towards enhanced antigen sensitivity and/or variant epitope recognition 10.

An alternative approach to TCR engineering relies on the generation of new specificities from naturally occurring TCR sequences, exemplified by the construction of hybrid αβ TCR pairs specific for HIV‐1 variants 20, 21. This strategy is enabled by a huge TCR clonotype bank, estimated to exceed 2 × 10^7^ unique receptors in any given individual 22, 23, 24. Moreover, it is established that mixed dimers can form between endogenous and introduced TCRs in primary human T cells, generating unknown neo‐reactivities 6. Although recent studies have implicated the variable domains and CDR3 loops as primary determinants of αβ TCR pairing 25, 26, 27, however, our current understanding is not sufficient to predict the impact of hybridization on antigen specificity and cross‐reactivity.

Despite the immense recombinatorial potential within the TCR repertoire, identical or closely related receptors specific for any given epitope are observed frequently in multiple individuals sharing a major histocompatibility complex (MHC) restriction molecule 24, 28. These public or near‐public TCRs provide template libraries for α and β chains with defined specificities. Hybrid combinations derived from such libraries may therefore allow the generation of TCRs with therapeutically desirable properties, such as improved antigen sensitivity and cross‐reactivity against naturally occurring epitope variants *in vivo* 10.

In the present study, we characterized TCR clonotypes specific for the immunodominant human leucocyte antigen (HLA)‐B*35:01‐restricted HIV‐1 negative regulatory factor (Nef)‐derived epitope VY8 (VPLRPMTY, residues 74–81). Although closely related TCR pairs were identified across multiple individuals, small differences in the primary amino acid sequence were found to exert dramatic phenotypic effects in terms of surface expression, antigen specificity and cross‐reactivity. Hybridization was also shown to confer neo‐specificities for variant antigens, an observation with therapeutic implications for highly mutable viruses.

## Materials and methods

### Cell culture

The TCR‐deficient mouse T cell hybridoma cell line TG40 (kindly provided by Takashi Saito, RIKEN Institute, Yokohama, Japan) was maintained in RPMI‐1640 containing 10% heat‐inactivated fetal calf serum (FCS) (Life Technologies, Carlsbad, CA, USA). Primary CD8^+^ T cells were maintained in RPMI‐1640 supplemented with 100 U/ml penicillin, 100 mg/ml streptomycin, 2 mM L‐glutamine and 10% heat‐inactivated FCS (all Life Technologies), together with 2.5% Cellkines (Helvetica Healthcare, Geneva, Switzerland), 200 IU/ml IL‐2 (PeproTech, Rocky Hill, NJ, USA) and 25 ng/ml IL‐15 (PeproTech). This study was approved by the relevant Institutional Review Boards.

### Flow cytometry

Tetrameric complexes of VY8/HLA‐B*35:01 (VY8/B35) conjugated to phycoerythrin (PE) or allophycocyanin (APC) (Molecular Probes/Life Technologies) were prepared as described previously 29, 30. Cells were labelled with the VY8/B35 tetramer for 20 min at 4°C and then stained with monoclonal antibodies (mAbs) as indicated for 20 min at 4°C. After two washes in phosphate‐buffered saline (PBS) containing 2% FCS, cells were fixed in 1% paraformaldehyde and acquired using a fluorescence activated cell sorter (FACS)Canto II flow cytometer (BD Biosciences, San Jose, CA, USA). Data were analysed with FlowJo software (Treestar Inc., Ashland, OR, USA). The following mAbs were used: (i) anti‐human CD3‐fluorescein isothiocyanate (FITC) and anti‐human CD8‐APC (DakoCytomation, Glostrup, Denmark); (ii) anti‐human CD8‐peridinin chlorophyll protein (PerCP) (BD Biosciences); and (iii) anti‐rat CD2‐PE and anti‐mouse CD3*ε*‐PE (Biolegend, San Diego, CA, USA). Dead cells were excluded from the analysis using 7‐aminoactinomycin D (7‐AAD) (Biolegend).

### Analysis of TCR‐encoding genes

TCR transcripts were amplified using a SMART PCR cDNA Synthesis Kit (Clontech/Takara Bio Inc., Shiga, Japan) and analysed via the ImMunoGeneTics database (http://www.imgt.org/), as described previously 29, 31.

### TCR reconstruction and analysis

Full‐length TCR‐α and ‐β constructs were cloned into the retroviral vector pMX incorporating *gfp* as a marker (kindly provided by Toshio Kitamura, University of Tokyo, Japan) and transduced into TCR‐deficient TG40 and TG40/CD8 cells, as described previously 29, 30, 32, 33. Transductants were identified by staining with anti‐mouse CD3*ε*‐PE, and brightly labelled cells were cloned by limiting dilution. Assays of T cell sensitivity were performed as described previously [30]. Briefly, C1R‐B*35:01 cells (10^4^ per well) and TCR‐transduced TG40/CD8 cells (2 × 10^4^ per well) were incubated with peptides as indicated for 24 h at 37°C. Supernatant was then analysed for interleukin (IL)‐2 content by measuring the proliferative activity of the indicator cell line CTLL‐2. Cross‐reactivity was assessed against a library of variant 11mer peptides (RPQVPLRPMTY) containing the VY8 index sequence (VPLRPMTY). In the VY8 index sequence, each position was substituted with every amino acid except cysteine to generate a total of 144 variant peptides, as described previously 34. Synthesized peptides were analysed for purity by mass spectrometry and high‐performance liquid chromatography (HPLC).

### TCR expression in primary CD8^+^ T cells

Full‐length TCR‐α and ‐β constructs were cloned into the lentiviral vector pELN (kindly provided by James L. Riley, University of Pennsylvania, Philadelphia, USA), incorporating a pair of 2A self‐cleaving peptides separating the TCR chains and rat CD2 (rCD2) as a marker for delivery into primary CD8^+^ T cells 10. Briefly, CD8^+^ T cells were isolated from HLA‐B*35^+^ healthy donor peripheral blood mononuclear cells (PBMCs) and stimulated with anti‐CD3/anti‐CD28 beads (Invitrogen, Carlsbad, CA, USA) for 24 h before transduction. Transductants were enriched using magnetic beads to yield rCD2^+^ populations with >90% purity. Assays of T cell sensitivity were performed as described previously 35. Briefly, TCR‐transduced CD8^+^ T cells (3 × 10^4^ per well) were mixed with peptide‐pulsed C1R‐B*35:01 cells (6 × 10^4^ per well) and incubated overnight at 37°C. Supernatant was then analysed for macrophage inflammatory protein (MIP)‐1β content by enzyme‐linked immunosorbent assay (ELISA), according to the manufacturer's instructions (R&D Systems, Minneapolis, MN, USA). Background values in the absence of peptide were subtracted.

### Octamer combinatorial peptide library scan

An octamer combinatorial peptide library (CPL) comprising 2.4 × 10^10^ different peptides (PepScan, Lelystad, the Netherlands) was divided into 160 submixtures in positional scanning format, as described previously 36, 37, 38. Target C1R‐B*35:01 cells (6 × 10^4^ per well) were preloaded with CPL submixtures (100 µg/ml) and incubated with effector T cells (3 × 10^4^ per well) overnight at 37°C. Supernatant was then analysed for MIP‐1β content by ELISA, as described previously 35, 37, 38, 39. The relative T cell response is shown as fold change MIP‐1β production with respect to the lowest response at each position. A fold response >1.5 was considered positive.

## Results

### TCR analysis of VY8‐specific CD8^+^ T cells

Molecular analysis of CD8^+^ T cell clones specific for the HIV‐1 Nef‐derived epitope VY8 (VPLRPMTY, residues 74–81) presented by HLA‐B*35:01 3, 38, 40, which is typically immunodominant in the acute/early phase of HIV‐1 infection 40, 41, showed that the TCR‐αβ variable segments were dominated by AV1 and BV11 in three different HIV‐1‐infected subjects (Pt‐01, Pt‐19 and Pt‐33) (Table 1). Similar bias was observed in VY8‐specific CD8^+^ T cell lines derived from two additional HLA‐B*35:01^+^ HIV‐1‐infected subjects (Supporting information, Fig. S1). Four TCR‐α chains exhibited highly similar CDR3α sequences, which varied at a single amino acid position incorporating Arg, Thr or Ser (designated A2‐R, A1‐R, A1‐T and A1‐S, respectively) (Table 1). Two of these TCR‐α chains shared an identical CDR3α sequence combined with the related variable segments AV1‐1 or AV1‐2 (designated A1‐R and A2‐R) (Table 1). Three TCR‐β chains with a common BV11 variable segment (designated B11, B11‐J1 and B11‐J2) displayed a conserved amino acid sequence across the CDR3β loop (xDI/LVxxE), whereas one TCR‐β chain expressed a BV4‐2 variable segment (designated B4) in conjunction with a long CDR3β sequence rich in alanine and aspartic acid residues (Table 1).

**Table 1:** T cell receptor (TCR)‐αβ chains used in this study

	Designation	V gene	J gene	CDR3 sequence	T cell	Subject
TRA	A2‐R	AV1‐2*01	AJ26*01	CAV RDNYGQN FVF	Clone‐S1	Pt‐33
	A1‐R	AV1‐1*01	AJ26*01	CAV RDNYGQN FVF	Line‐100	Pt‐100
	A1‐T	AV1‐1*01	AJ26*01	CAV TDNYGQN FVF	Clone‐139	Pt‐19
	A1‐S	AV1‐1*01	AJ26*01	CAV SDNYGQN FVF	Line‐100	Pt‐100
	A1‐J31	AV1‐1*01	AJ31*01	CAV TSGDAR LMF	Clone‐H127	Pt‐01
		AV1‐2*01	AJ31*01	CAV EGDNAR LMF	Clone‐H218	Pt‐01
		AV1‐1*01	AJ31*01	CAV RDGGNNAR LMF	Clone‐136	Pt‐19
TRB	B11	BV11‐1*01	BJ2‐7*01	CASS SDIVSYE QYF	Clone‐H127	Pt‐01
	B11‐J1	BV11‐2*01	BJ1‐1*01	CASS LDLVSTE AFF	Clone‐139	Pt‐19
	B11‐J2	BV11‐2*01	BJ2‐1*01	CASS PDLVDNE QFF	Line‐100	Pt‐100
	B4	BV4‐2*01	BJ2‐3*01	CASS QAADAAITDADT QYF	Clone‐S1	Pt‐33
		BV4‐2*01	BJ2‐1*01	CASS PEVAANNE QFF	Clone‐H218	Pt‐01
		BV7‐2*03	BJ2‐1*01	CASS PTPQGDYE QFF	Clone‐136	Pt‐19

Underlining in CDR3α and CDR3β represents amino acid differences and conserved amino acids, respectively. A1‐T/B11‐J1, A1‐J31/B11 and A2‐R/B4 are naturally isolated TCR pairs from clones 139 (Pt‐19), H127 (Pt‐01) and S1 (Pt‐33).

### Functional reconstruction of naturally isolated TCR pairs

**Figure 1:**
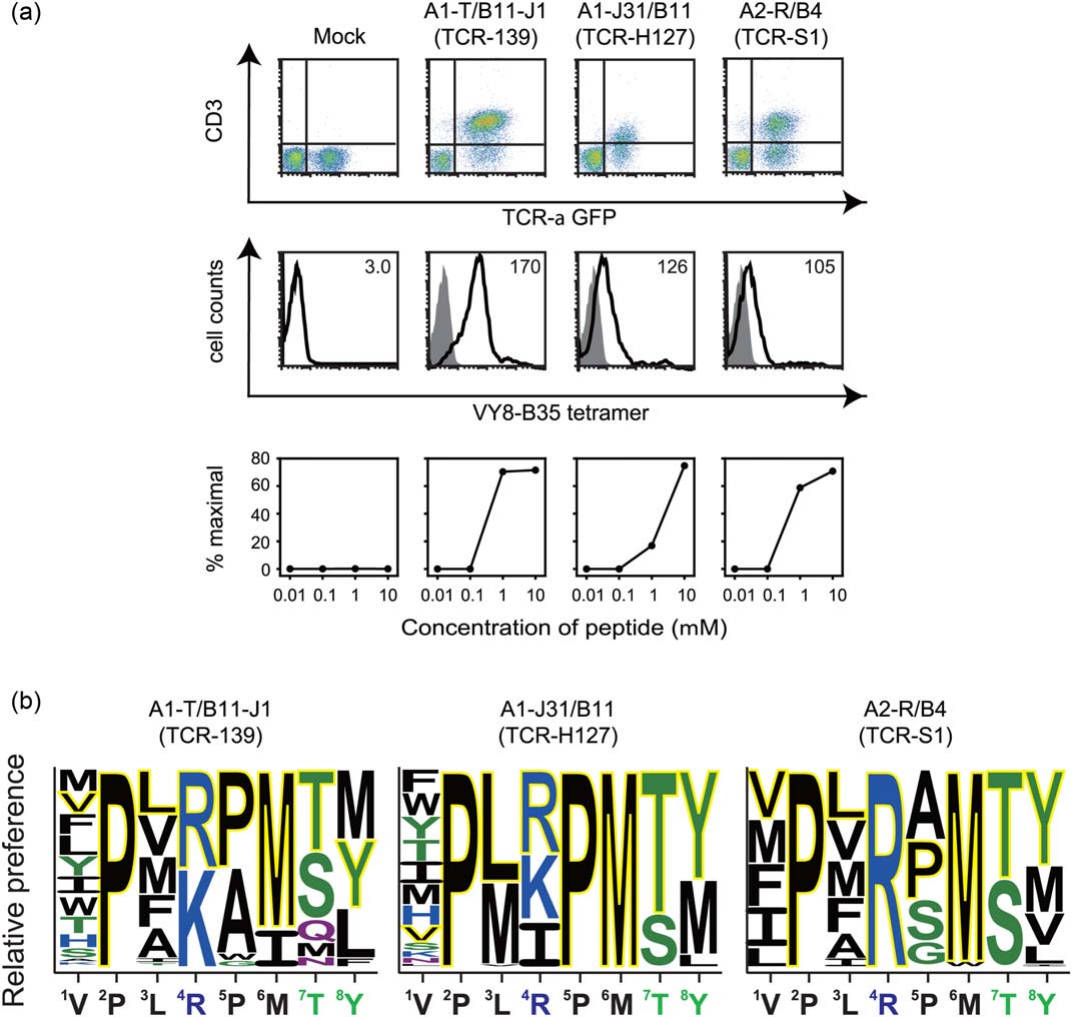
Reconstruction of naturally isolated T cell receptor (TCR) pairs. (a) TCR‐deficient TG40 cells expressing the naturally isolated αβ TCR pairs A1‐T/B11‐J1 (TCR‐139), A1‐J31/B11 (H127) or A2‐R/B4 (S1) were stained with anti‐CD3 monoclonal antibody (mAb) (top row) or VY8/B35 tetramer (middle row) and analysed by flow cytometry. Mock‐transduced TG40 cells were used as negative controls. Tetramer staining is depicted as mean fluorescence intensity after gating on 7‐aminoactinomycin D (7‐AAD)^–^ green fluorescent protein (GFP)^+^CD31^+^ cells. To assess T cell sensitivity (bottom row), TCR genes were delivered into TG40 cells expressing human CD8α, as described previously 34. CD3^+^ cells were then enriched using magnetic beads and analysed for interleukin (IL)‐2 secretion in response to various concentrations of VY8 peptide. The amount of IL‐2 obtained with mock‐transduced TG40 cells was consistently <5.0 U/ml and the amount of IL‐2 secreted by TCR‐transduced TG40 cells was expressed as % maximal, normalized with respect to the anti‐CD3 mAb‐mediated activation. Data are representative of duplicate assays. **(b)** TCR cross‐reactivity was evaluated against a library of peptides in which each position was substituted with every amino acid except cysteine to generate a total of 144 individual variants (5 µM per peptide). Antigen sensitivity was calculated as the percent of maximum response compared to anti‐CD3 mAb‐stimulated cells. Graphical representations showing relative preferences for amino acid residues at each position were generated using WebLogo 3 (http://weblogo.threeplusone.com/) 38. Colours represent physicochemical properties: green, polar (G, S, T, Y and C); purple, neutral (Q and N); blue, basic (K, R and H); red, acidic (D and E); black, hydrophobic (A, V, L, I, P, W, F and M). The index residue at each position is outlined in yellow. Residue size is proportional to T cell recognition preference.

The TCR‐deficient T cell line TG40 has been used previously to examine TCR–peptide MHC (pMHC) interactions 29, 30, 34 and characterize murine and/or human TCR‐αβ pairs 29, 31, 32, 33, 42. To test integrity in this system, TG40 cells were transduced with retroviral vectors expressing three TCR pairs (TCR‐139, H127 and S1 incorporating A1‐T/B11‐J1, A1‐J31/B11 and A2‐R/B4, respectively) isolated from CD8^+^ T cell clones specific for VY8 (Table 1). All three TCRs were expressed on the cell surface. In each case, transduced T cells bound the VY8/B35 tetramer and exhibited VY8‐specific IL‐2 production (Fig. 1a), confirming specificity and functionality.

The cross‐reactivity profiles of these naturally isolated TCR pairs were examined using a set of peptides containing substitutions with every amino acid except cysteine at each of the eight positions along the peptide backbone (a total of 144 variant peptides) 34. Graphical representations were used to display the unique preferential residue recognition signature of each TCR‐α and ‐β pair (Fig. 1b). All TCR pairs showed a similar cross‐reactivity profile, recognizing multiple amino acids at P1, but only limited numbers of substitutions at P2, P4 and P6 (Fig. 1b). However, TCR‐139 and S1 recognized a wider range of hydrophobic residues such as Leu, Val, Met, Phe and Ala at P3, whereas H127 recognized only Leu and Met at this position (Fig. 1b). These results suggest that individual TCR‐α and ‐β chains can confer unique antigen recognition profiles.

### Cell surface expression and epitope specificity of hybrid TCR pairs

**Figure 2:**
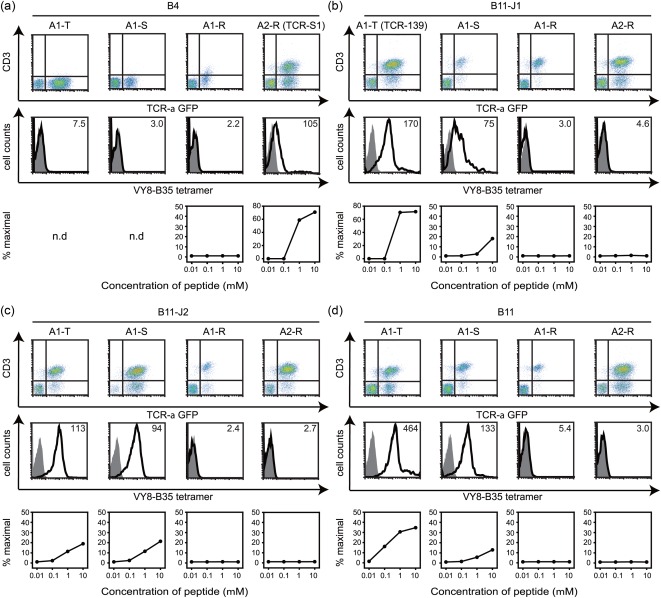
Reconstruction of hybrid T cell receptors (TCRs). (a–d) The indicated combinations of TCR‐α (A1‐T, A1‐S, A1‐R and A2‐R) and ‐β (B4, B11‐J1, B11‐J2 and B11) chains were analysed for cell surface expression of TCR/CD3, VY8/B35 tetramer binding and VY8 reactivity, as described in Fig. 1a.

To test the antigen specificity of hybrid TCR pairs, TG40 cells were transduced with retroviral vectors expressing 16 different combinations of VY8‐specific TCR‐α and ‐β chains (Table 1). Cell surface TCR/CD3 expression was detected for 14 of 16 hybrid pairs, only seven of which showed VY8/B35 tetramer binding and VY8‐specific IL‐2 production (Fig. 2). Three TCR‐α chains with a single amino acid difference in the CDR3α loop (A1‐T, A1‐S and A1‐R) were expressed at the cell surface in complex with all TCR‐β chains except B4 (Fig. 2a). None of the TCR pairs with A1‐R showed VY8 specificity. Of note, tetramer binding intensity and antigen sensitivity did not correlate for some hybrid TCRs, such as A1‐T/B11 and A1‐S/B11 (Fig. 2d).

Next, we examined the impact of AV1‐1 and AV1‐2 on TCR expression and antigen specificity. The backbone framework of these variable segments differs at 17 of 128 amino acid positions (ImMunoGeneTics database; http://www.imgt.org/). The A1‐R and A2‐R TCR‐α chains also exhibit amino acid differences in the CDR1α (TSGFYG and TSGFNG) and CDR2α (NALDGL and NVLDGL) loops. Although A2‐R/B4 was expressed at the cell surface and displayed VY8 specificity comparable to other naturally isolated TCR pairs, substitution of the TCR‐α chain with A1‐R substantially decreased TCR/CD3 surface expression and abrogated VY8 specificity (Fig. 2a). In addition, other TCR‐α chains (A1‐T and A1‐S) failed to express in combination with B4 (Fig. 2a). These results suggest that the germline‐derived sequence contributes to functional TCR‐αβ heterodimer formation and T cell specificity.

### Cross‐reactivity profiles of hybrid TCR pairs

**Figure 3:**
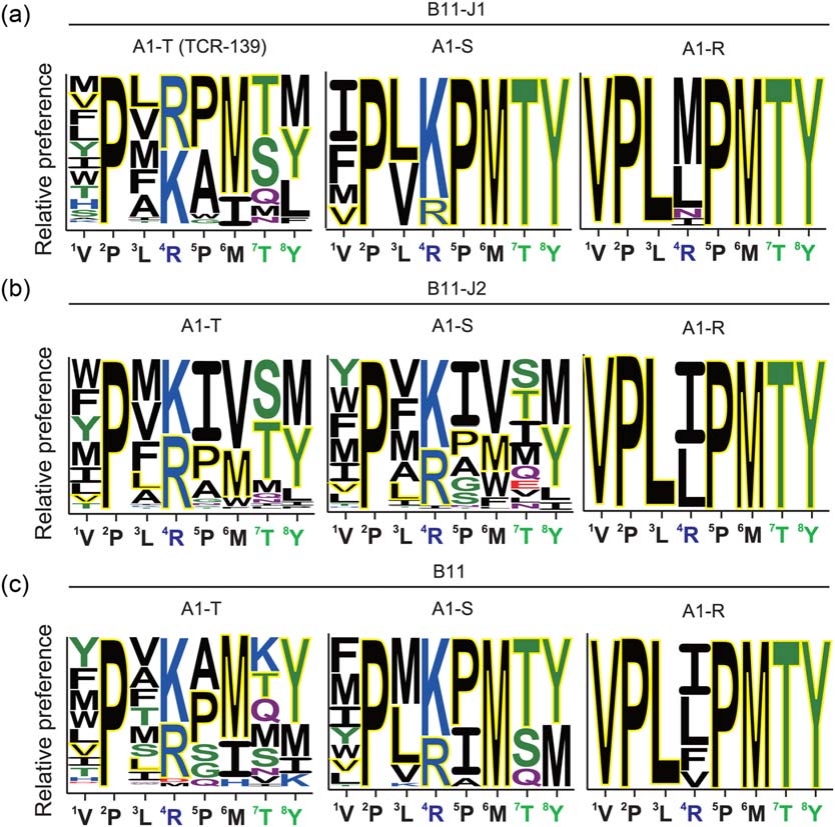
Cross‐reactivity of hybrid T cell receptors (TCRs). (a–c) The indicated combinations of TCR‐α (A1‐T, A1‐S and A1‐R) and ‐β (B11‐J1, B11‐J2 and B11) chains were analysed for cross‐reactivity, as described in Fig. 1b.

Next, we tested the cross‐reactivity profiles of hybrid TCR pairs against variant peptides. Single amino acid differences in the CDR3α loop and TCR‐αβ pairing substantially affected fine specificity (Fig. 3). The A1‐T chain showed a similar cross‐reactivity profile combined with B11‐J1 (a naturally isolated TCR pairing), B11‐J2 or B11 (Fig. 3a–c). In contrast, the CDR3α Thr to Ser mutation (A1‐T to A1‐S) dramatically decreased cross‐reactivity at P1, P3 and P7 when combined with B11‐J1 (Fig. 3a), an effect that was compensated by switching the TCR‐β chain to B11‐J2 or B11 (Fig. 3b,c). Although positively charged residues (Lys and Arg) at P4 were required for recognition by various VY8‐specific CD8^+^ T cell clones 38, as well as most of the hybrid pairs bearing A1‐T but not A1‐S (Figs. 1 and 3), TCRs with the A1‐R chain in combination with any of the three TCR‐β chains favoured hydrophobic residues (Ile and Leu) at this position (Fig. 3). These data explain why TCRs incorporating A1‐R lose VY8 specificity (Fig. 2), and suggest that clonotypically similar hybrid receptors can acquire neo‐variant specificities.

### Hybrid TCR expression, specificity and cross‐reactivity in primary CD8^+^ T cells

**Figure 4:**
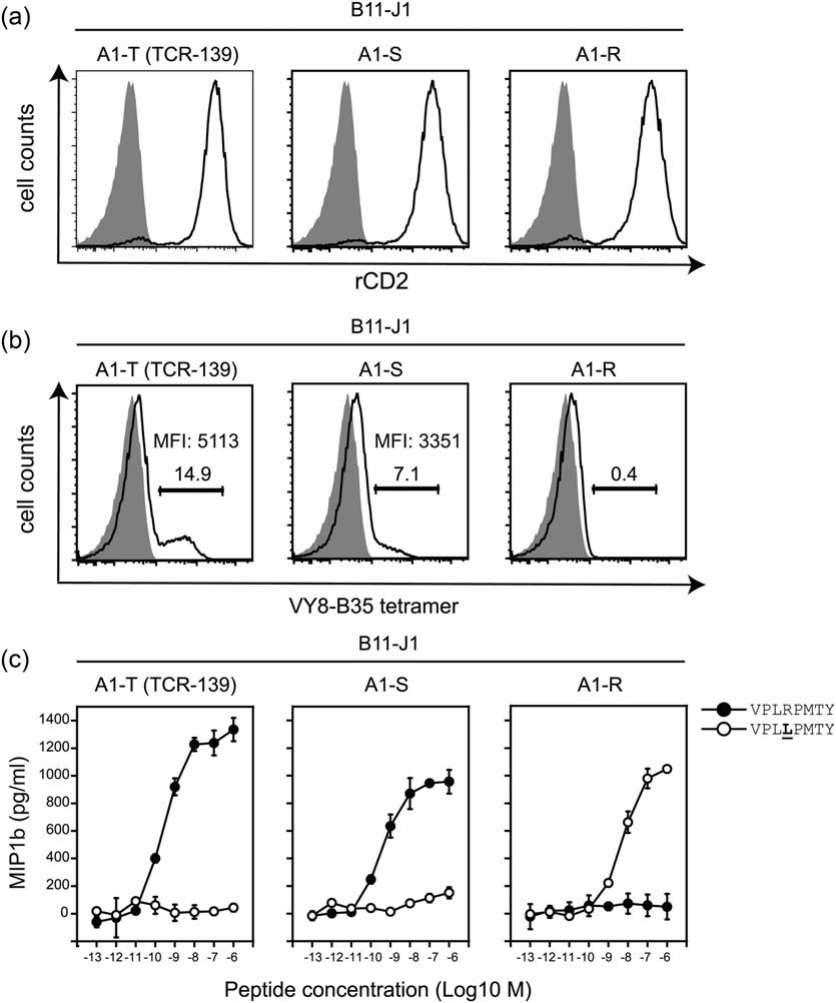
Reconstruction of hybrid T cell receptors (TCRs) in primary CD8^+^ T cells. (a–c) Primary CD8^+^ T cells transduced with the indicated TCR pairs were examined for rCD2 expression (a), VY8/B35 tetramer staining after gating on 7‐aminoactinomycin D (7‐AAD)^–^CD8^+^ cells (b) and macrophage inflammatory protein (MIP)‐1β production in response to peptide stimulation (c). In (a) and (b), data are representative of duplicate assays. In (b), the percentage and mean fluorescence intensity of live tetramer^+^ CD8^+^ T cells are indicated in each histogram. In (c), standard deviation from the mean of two replicates is shown.

To confirm the antigen specificity of T cells expressing hybrid TCR pairs, B11‐J1 receptors paired with A1‐T, A1‐S or A1‐R were introduced into primary CD8^+^ T cells and enriched on the basis of rCD2 expression to >90% purity (Fig. 4a). CD8^+^ T cells expressing the naturally isolated TCR pair A1‐T/B11‐J1 stained efficiently with the VY8/B35 tetramer, confirming the integrity of TCR reconstruction (Fig. 4b). In contrast, CD8^+^ T cells expressing A1‐S stained moderately with the VY8/B35 tetramer and those expressing A1‐R stained poorly (Fig. 4b), confirming the data obtained using TG40 cells (Fig. 2). Moreover, CD8^+^ T cells expressing A1‐T and A1‐S, but not A1‐R, recognized target cells pulsed with the index VY8 peptide (Fig. 4c). Conversely, CD8^+^ T cells expressing A1‐R displayed a unique reactivity against the variant peptide with Leu at P4, which was not recognized by cells expressing A1‐T or A1‐S (Fig. 4c). Again, these patterns are consistent with the data generated using TG40 cells (Figs 2b, 3a).

**Figure 5:**
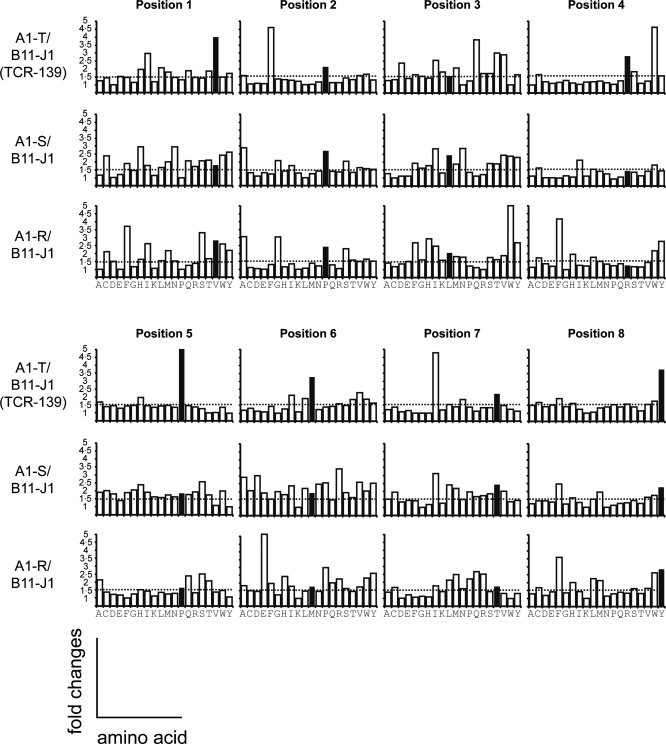
Cross‐reactivity of hybrid T cell receptors (TCRs) in primary CD8^+^ T cells. Primary CD8^+^ T cells expressing hybrid TCRs were tested for preferred recognition residues at each position along the peptide backbone using an octamer combinatorial peptide library incorporating a total of 2.4 × 10^10^ different octamer peptides. In each of 160 peptide submixtures, one position was fixed with a defined amino acid residue and all other positions were degenerate, including any amino acid except cysteine. Macrophage inflammatory protein (MIP)‐1β release was quantified by enzyme‐linked immunosorbent assay (ELISA). Responses are shown as fold change MIP‐1β production relative to the lowest response at each position defined as 1.0. Background responses were 70 ± 8.9 pg/ml and the lowest response for CD8^+^ T cells expressing A1‐T, A1‐S and A1‐R at each position were 183 ± 26, 93 ± 44 and 122 ± 19 pg/ml, respectively. Fold changes >1.5 were considered positive. A representative set of duplicate assays is shown. Black bars depict residues corresponding to the VY8 index sequence.

To clarify these changes in antigen specificity towards not only substitutions within the VY8 peptide backbone but also towards all possible amino acid combinations of the octamer peptide, we employed a library containing 2.4 × 10^10^ different octamer peptides, which enabled qualitative mapping of preferred T cell recognition residues at each position along the peptide backbone 38 (Fig. 5). Primary CD8^+^ T cells expressing A1‐T recognized the index amino acid at each position, consistent with previous observations 38. Conversely, CD8^+^ T cells expressing A1‐S or A1‐R showed different patterns of cross‐reactivity with reduced recognition of the index Arg residue at P4 as well as other amino acids, such as Pro at P5 and Met at P6 (Fig. 5). These data further support the notion that closely related hybrid TCRs can display substantial differences in fine specificity.

## Discussion

In this study, we artificially paired α and β chains from clonotypically similar TCRs specific for the HLA‐B*35:01‐restricted HIV‐1 Nef‐derived epitope VY8 to examine the impact of recombinatorial sequence diversity on receptor expression, antigen specificity and cross‐reactivity. The key findings were as follows: (i) some hybrid TCRs failed to express at the T cell surface despite close homology with naturally isolated αβ chain combinations; (ii) approximately 50% of those αβ TCRs that expressed lost specificity for the index VY8 peptide sequence; and (iii) allelic differences between AV1‐1 and AV1‐2, and an amino acid change in the CDR3α loop, not only altered surface TCR/CD3 levels, index peptide recognition and cross‐reactivity profiles, but also generated neo‐specificities for variant antigens. These data demonstrate that even small differences between paired α and β chain sequences generated naturally in response to a given antigen can dramatically influence the physical and functional properties of the resultant TCR.

Public or near‐public TCRs have been identified in antigen‐specific CD8^+^ T cell responses to variable pathogens such as HIV and simian immunodeficiency virus (SIV) 24, 28, and the highly focused nature of these biased repertoires can facilitate immune escape under certain conditions 43, 44. Accordingly, we hypothesized that hybrid TCRs with intact surface expression and HIV‐1‐derived epitope specificity could be constructed by shuffling such closely related α and β chains. Of 16 αβ combinations, however, only seven pairs generated TCRs that retained cell surface expression and index epitope specificity. In addition, some hybrid TCRs that lost reactivity against the index epitope displayed neo‐specificities for variant peptides. These results demonstrate that clonotypically similar TCRs are partner chain‐dependent and cannot be considered functionally equivalent. Structural approaches are warranted to determine how allelic differences and an amino acid change in the CDR3α loop alter TCR pairing and specific antigen contacts at the molecular interface.

In broad terms, the CDR1 and CDR2 loops predominantly engage the presenting MHC molecule, whereas the CDR3 loops are typically orientated to interact primarily with the bound peptide epitope 45, 46. The CDR3 loop is highly variable as a result of somatic V‐(D)‐J rearrangement and junctional diversification 47. In our analysis of naturally arising VY8‐specific TCRs, we identified closely‐related AV1‐1/AJ26 α chains that differed at a single position with Thr, Ser or Arg in the CDR3α loop. Shuffling studies revealed that both A1‐T and A1‐S retained VY8 specificity and showed similar TCR cross‐reactivity profiles in combination with different BV11 β chains (B11‐J1, B11‐J2 and B11) (Fig. 3). Similar findings have been reported previously, where specific α chains were able to pair productively with multiple β chains in the context of a single TCR specificity 25, 26, 32.

It is notable that the B4 β chain exhibited a long CDR3β loop and limited capacity to pair with alternate α chains (Fig. 2). This observation is consistent with a previous report showing the TCR‐αβ pairing being inhibited by a long CDR3 loop 27. Conversely, A2‐R only paired with the B4 β chain. Furthermore, A2‐R failed to recognize VY8 or any single‐substituted peptide variants thereof (*n* = 144) in partnership with B11‐J1, B11‐J2 or B11 (data not shown), whereas A1‐R paired with these β chains recognized variant peptides substituted at P4. Germline‐encoded differences in the α chain may therefore play a crucial role in TCR pairing, antigen specificity and cross‐reactivity. In support of this notion, a recent structural study showed that allelic variation (Gln and His) in public β chains (BV9‐1 and BV9‐2) specific for the HLA‐B*35:01‐restricted Epstein–Barr virus (EBV) EBNA‐1‐derived epitope HY11 (HPVGEADYFEY, residues 407‐417) affected charge complementarity at the TCR/pMHC interface 48.

Some limitations of our study merit mention. First, studies of TCR pairing and antigen specificity feature numerous challenges and limitations. For example, although a variety of human TCR‐αβ chains are physically and functionally reconstructed on the surface of murine‐derived cells 29, 30, 31, 32, 33, 42 as well as human T cells 25, 27, 33, 49, non‐physiological constraints on the hybrid TCR/CD3 complex may complicate data interpretation 50. Indeed, some human TCR‐αβ pairs such as A1‐T/B4 and A1‐S/B4 failed to express as TCR/CD3 on the TG40 cell surface in this study (Fig. 2a). However, the B4 β chain can be expressed as TCR/CD3 on the TG40 cell surface when combined with A2‐R and A1‐R (Fig. 1a), as well as other AV1‐2^+^ TCR‐α chains (data not shown), confirming the functionality of the TCR‐α and ‐β chains used in the study. Further studies are needed to elucidate comprehensively the molecular basis of the TCR‐αβ heterodimer assembly in the context of the TCR/CD3 complex on the cell surface 50. Second, prediction of specific recognition patterns from primary TCR sequence data is still a challenging task. Here, we examined a set of very similar TCR‐αβ pairs for antigen specificity and cross‐reactivity by using HLA tetramers, T cell stimulation assay, and combinatorial libraries. Although the results do not suggest common and shared pathway(s) for determination of TCR specificity and cross‐reactivity our data highlight, rather, that clonotypically similar TCRs can display profound differences in surface expression, antigen specificity and cross‐reactivity. These findings suggest that even minor recombinatorial variability can expand the composite functional capabilities of any given antigen‐specific TCR repertoire, potentially enabling enhanced immune control in the case of highly mutable viruses while avoiding detrimental cross‐reactivity or autoreactivity. Further investigations of a variety of TCRs specific for other pathogens and tumours are warranted.

## Disclosure

The authors declare no commercial or financial conflict of interest.

## Author contributions

C. M. and T. U. conceived and designed the study. C. M., J. S. B. and T. U. performed the experiments. C. M., J. S. B., D. A. P., A. K. S. and T. U. wrote the manuscript.

## Supporting information

Additional Supporting information may be found in the online version of this article at the publisher's Web site:

**Fig. S1.** T cell receptor (TCR) usage in VY8‐specific CD8^+^ T cell lines. (a, b) Usage of human T cell receptor alpha variable region (TRAV) (a) and human T cell receptor beta variable (TRBV) (b) segments in VY8‐specific CD8^+^ T lines generated from two human leucocyte antigen (HLA)‐B*35^+^ subjects infected with HIV‐1 (Pt‐100 and Pt‐168). Peripheral blood mononuclear cells (PBMCs) were stimulated with index peptide for 2 days and VY8/B35 tetramer^+^ CD8^+^ T cell populations were sorted by flow cytometry.
