# Supplementary figures and images for: Clonotypically similar hybrid αβ T cell receptors can exhibit markedly different surface expression, antigen specificity and cross‐reactivity
Source: Clin Exp Immunol. 2015 May 15;180(3):560–70. doi: 10.1111/cei.12610 (PMC4449784; doi:10.1111/cei.12610)

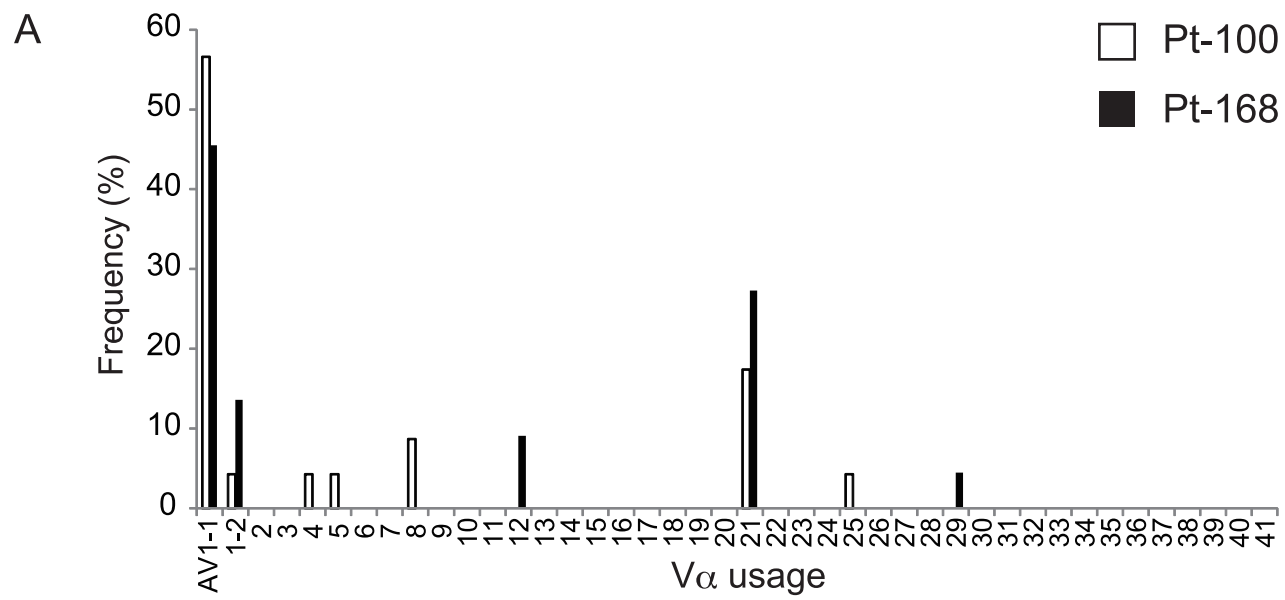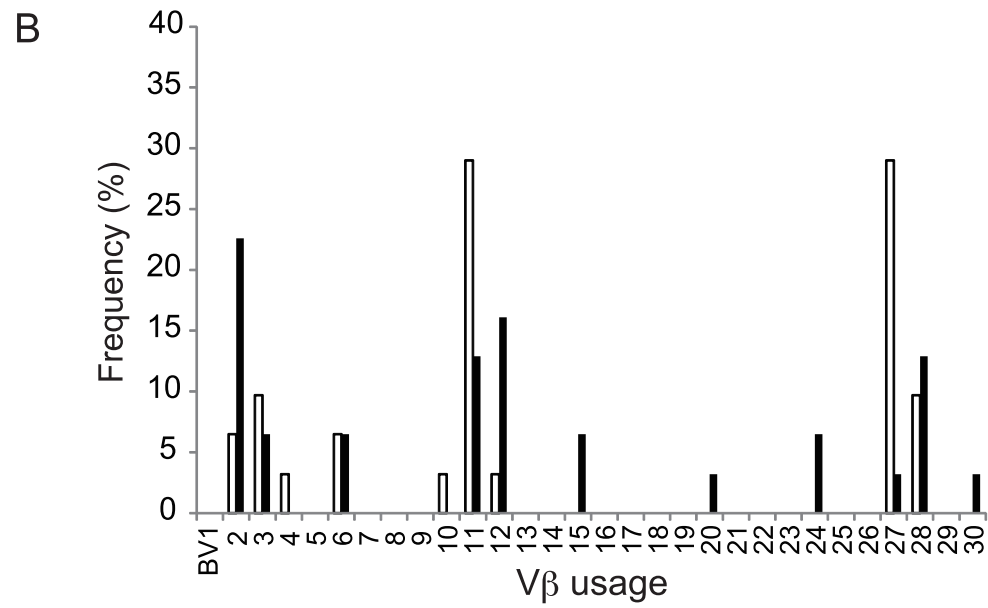

Supplemental Figure 1 Motozono et al

Supplement: Supplementary file 1 — Fig. S1. T cell receptor (TCR) usage in VY8‐specific CD8+ T cell lines. (a, b) Usage of human T cell receptor alpha variable region (TRAV) (a) and human T cell receptor beta variable (TRBV) (b) segments in VY8‐specific CD8+ T lines generated from two human leucocyte antigen (HLA)‐B*35+ subjects infected with HIV‐1 (Pt‐100 and Pt‐168). Peripheral blood mononuclear cells (PBMCs) were stimulated with index peptide for 2 days and VY8/B35 tetramer+ CD8+ T cell populations were sorted by flow cytometry. [file cei0180-0560-sd1.pdf]
